# A new species of *Monocheres* Stock (Copepoda, Siphonostomatoida, Asterocheridae) from shallow waters off Florida, USA: an unexpected discovery

**DOI:** 10.3897/zookeys.607.9137

**Published:** 2016-07-27

**Authors:** Eduardo Suárez-Morales

**Affiliations:** 1 El Colegio de la Frontera Sur (ECOSUR), Unidad Chetumal. A.P. 424, Av. Centenario Km 5.5, Chetumal, Quintana Roo 77014, Mexico

**Keywords:** Associated copepods, hesionid polychaetes, interstitial, taxonomy

## Abstract

The rare asterocherid copepod genus *Monocheres*, ectosymbionts of corals and sponges, contains only two species, one from Mauritius (Indian Ocean) and the other one from Brazil (western Atlantic). From the analysis of the digestive caecum contents of the benthic hesionid polychaete *Hesione picta* Müller, 1858, an adult female of an undescribed species of *Monocheres* was unexpectedly recovered; it is the third species of this rare asterocherid genus. The new species, *Monocheres sergioi* **sp. n.**, has the distinctive reduction of the fifth leg as a process with a single seta. It differs from its two other congeners by several characters including the presence of an inner basipodal spine, the armature details of the third exopodal segment of leg 1, the shape of the cephalosome and pedigerous somites 3 and 4, and the ornamentation of the postero-lateral corners of the genital double-somite. The main synapomorphies include the presence of spinules along the posterior margin of the first leg coxal sclerite and the reduced, spiniform coxal seta on leg 4. The biology and feeding habits of the polychaete containing this specimen suggests that the copepod was ingested as an ectosymbiont from sponges or coral but it is also possible that it was consumed from an ophiurid echinoderm. This finding allows an expansion of the genus geographical distribution in the northwestern Atlantic. A key to the species of *Monocheres* is also provided.

## Introduction

The copepod family Asterocheridae Giesbrecht, 1899 includes a highly diverse group of associated forms that have been recorded as ectosymbionts from a wide array of benthic invertebrates, including sponges, corals, ascidians, echinoderms, bryozoans, and mollusks (Boxshall and Halsey 2004). The type genus, *Asterocheres* Boeck, 1859 is clearly the most speciose in the family, and has more than 100 species (Kim 2010; Walter and Boxshall 2016). Several of the smallest asterocherid genera include only one or a few species that are rarely found. One of these groups is *Monocheres* Stock, 1966; it contains only two species. The first one, *Monocheres mauritianus* Stock, 1966 was described from specimens obtained from corals collected in Mauritius, Indian Ocean (Stock 1966). More than 30 years later, a second species (*Monocheres cagarrensis* Johnsson & Bustamante, 1997) was described from sponges in Brazilian islands off Rio de Janeiro (Johnsson and Bustamante 1997). This is a very unusual asterocherid genus, whose distinctive character is the strongly reduced fifth leg, represented by a papilla-like process fused to the pediger lateral margin and armed with a single distal seta (Stock 1966).

During a biological survey of the benthic invertebrates in the Florida Keys, benthic polychaetes of the genus *Hesione* were obtained. The taxonomical analysis of the genus includes the morphology of the enteric caeca, elongate internal sac-like structures. During the examination of dissected caeca of an individual of *Hesione picta* Müller, 1858, both a nereid polychaete and surprisingly, a copepod were found. The specimen was given to me for taxonomic analysis resulting in the identification of an undescribed species of *Monocheres*. In this report this specimen is described in full and compared with the other two known species of the genus; a key for the identification of the species of *Monocheres* is also provided.

## Methods

The polychaete from which the copepod was extracted was obtained by hand during sampling dives in shallow littoral areas off Long Key, Florida Keys, Florida. The copepod specimen was transferred to glycerol and lightly stained with Methylene Orange for taxonomical analysis. The specimen was observed and analyzed in whole and then dissected with sharpened needles; the appendages were examined as temporary mounts in glycerin and later sealed with Entellan® as permanent mounts. Drawings were prepared using a camera lucida mounted on an E-200 Nikon compound microscope with Nomarski DIC at magnifications of 400 and 1000×. Terminology of the body parts and appendages followed Huys and Boxshall (1991); abbreviations used in this work are: EXP = exopod, ENP = endopod. Body length of the copepod was measured from the anterior margin of the rostrum to the posterior margin of the caudal rami. The polychaete and the copepod are deposited in the collection of the Florida Museum of Natural History (FLMNH).

## Results

### Order Siphonostomatoida Thorell, 1859 Family Asterocheridae Giesbrecht, 1899 Genus *Monocheres* Stock, 1966

#### Monocheres sergioi sp. n.

Taxon classificationAnimaliaSiphonostomatoidaAsterocheridae

http://zoobank.org/7FE268B2-295E-4317-8657-716A6EF50478

##### Material examined.

**Figure 1.**
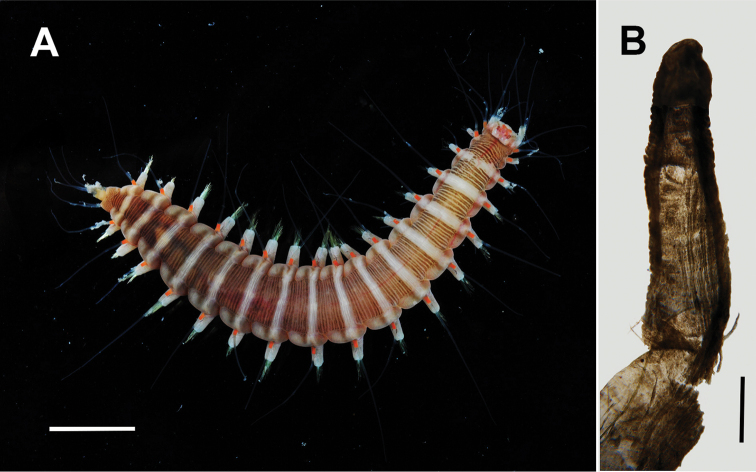
Specimen of *Hesione picta* Müller containing the copepod *Monocheres sergioi* sp.n. from off Long Key, Florida. **A** habitus, dorsal view **B** dissected digestive caecum. Scale bars: **A** = 5 mm, **B** = 1 mm. Photograph **A** by FLMNH-IZ team, **B** by Sergio Salazar-Vallejo.

Holotype. One adult female from a digestive caecum (Fig. 1A) of the hesionid polychaete *Hesione picta* Müller, 1858 (see Hartman, 1959) (UF 1594, KEYS-0778) collected in Monroe County, Florida Keys, Long Key, LONF1 tower dive site, W of Florida Keys Marine Laboratory (24.844°N, 80.864°W), at depth of 2 m, by Gustav Paulay. Body length of polychaete 28 mm long, 5 mm wide, 16 chaetigers (Fig. 1B, C).

##### Diagnosis.

Asterocherid with reduced fifth leg, represented by low protuberance armed with single distal seta. Genital double-somite with acute chitinous projection on posterolateral corners. Pediger 1 with posterolateral corners rounded, not produced, pediger 3 with posterior margin weakly curved. Anal somite with crenulated posterior margin. First segment of antennary endopod shorter than basis. Coxal sclerite with spinules. Coxal seta on leg 4 reduced, spiniform, third exopod of leg 4 with four spines, shorter than segmental width.

##### Description of adult female holotype.

**Figure 2.**
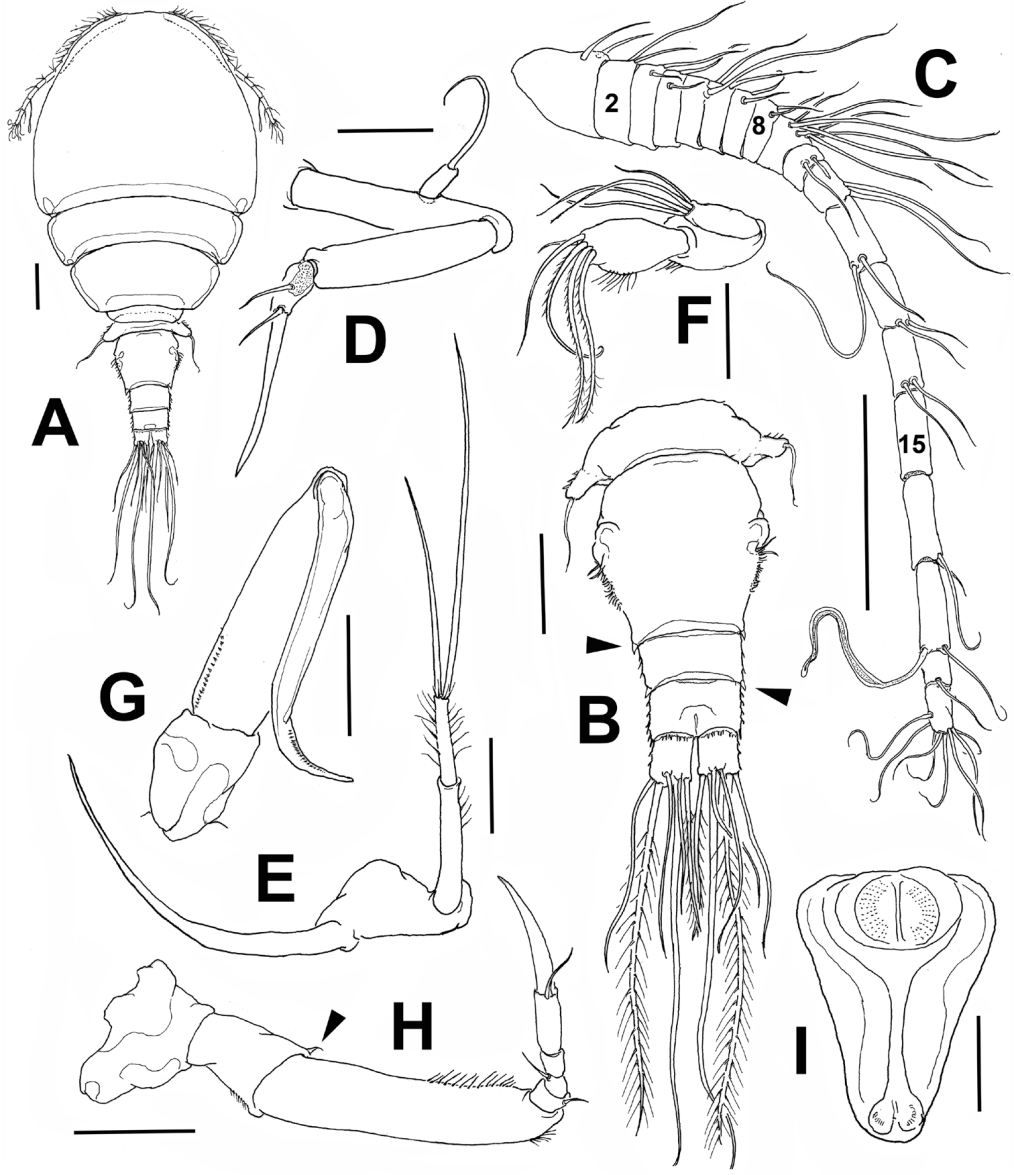
*Monocheres sergioi* sp. n. from Florida. Holotype female. **A** habitus, dorsal view **B** fifth pedigerous somite and urosome, dorsal view **C** antennule **D** antenna **E** mandible **F** maxillule **G** maxilla **H** maxilliped with minute basal seta arrowed **I** oral cone, ventral view. Scale bars: **A–C** = 100 µm, **D–F, I** = 20 µm, **G, H** = 50 µm.

Total body length from anteriormost end of cephalosome to posterior margin of caudal rami: 998 µm. Body (Fig. 2A) robust, with broad, rounded prosome, body widest at first pedigerous somite, slightly flattened dorsoventrally. Length ratio of prosome/ urosome = 2.2. First pedigerous somite with leg 1 completely fused to cephalosome. Pedigerous somites gradually tapering posteriorly. Pedigerous somite 4 narrowest, partially covered by third pedigerous somite in dorsal view. Posterolateral corners of pedigerous somites 1–3 rounded, lacking processes. Fifth pedigerous somite wider than fourth. Urosome 254 µm long, with three somites, genital double-somite 170 µm long barrel-shaped, slightly longer than wide, representing 67% of urosome (Fig. 2B). Genital openings located dorsolaterally, at widest section of somite, with adjacent row of short setules and low, rounded integumental expansion. Postero-lateral corners of genital double-somite smooth. Preanal somite subrectangular, 45 µm long, slightly shorter than succeeding anal somite (51 µm), both ornamented with spinules on lateral margin. Caudal rami 43 µm long, slightly shorter than anal somite; armed with 6 setae. Innermost terminal seta 130 µm, outermost terminal seta 185 µm, inner dorsal seta 134 µm, outer dorsal seta 167 µm, two long, relatively thicker median terminal setae, outer 315 µm and inner seta 338 µm.

*Antennule* (Fig. 2C) 392 µm long, excluding setae; 19-segmented. Segmentation (between brackets), segmental homologies (Roman numerals), and setation (s=setae, ae= aesthetascs) as follows: (1)I-2s, (2)II-2s, (3)III-2s, (4)IV-2s, (5)V-2s, (6)VI-2s, (7)VII-2s, (8)VIII-2s, (9)IX-XIII-7s, (10)XIV-2s, (11)XV-2s, (12) XVI-2s, (13)XVII-2s, (14)XVIII-2s, (15)XIX-0, (16)XX-2s, (17)XXI-ls+ae, (18)XXII-XXIII-1s, (19)XXIV-XXVIII-8.

*Antenna* (Fig. 2D) with slender, elongate basis carrying short, 1-segmented exopod and long, well-developed endopod. EXP longer than wide, armed with one long seta. ENP1 slightly shorter than basis. ENP2 armed with 1 seta, ENP3 longer than second, armed with short seta and stout, slightly curved terminal claw.

*Oral cone* (Fig. 2I) with usual structure of asterocherids, produced into siphon-like distal portion, reaching insertion of leg 1.

*Mandible* (Fig. 2E) consisting of long, slender stylet and 2-segmented palp, bearing 2 unequal apical setae; palp segments ornamented with setules.

*Maxillule* (Fig. 2F) bilobed, consisting of short, narrow outer lobe, armed with 4 subequally long distal setae, and wider, medially inflate and longer inner lobe, ornamented with row of short setules, bearing 4 long and 1 short pinnate setae.

*Maxilla* (Fig. 2G) two-segmented, including short subrectangular proximal syncoxa and distal elongate basis, longer than proximal segment, with row of small spinules proximally. Distally curved basipodal claw ornamented with spinules.

*Maxilliped* (Fig. 2H) consisting of syncoxa, subrectangular basis and 4-segmented endopod; syncoxa unarmed, basis with minute inner seta and row of short spinules on distal outer margin. ENP segments armed with 1, 1, 0, and 1 setae, respectively; terminal claw thick, weakly curved.

**Figure 3.**
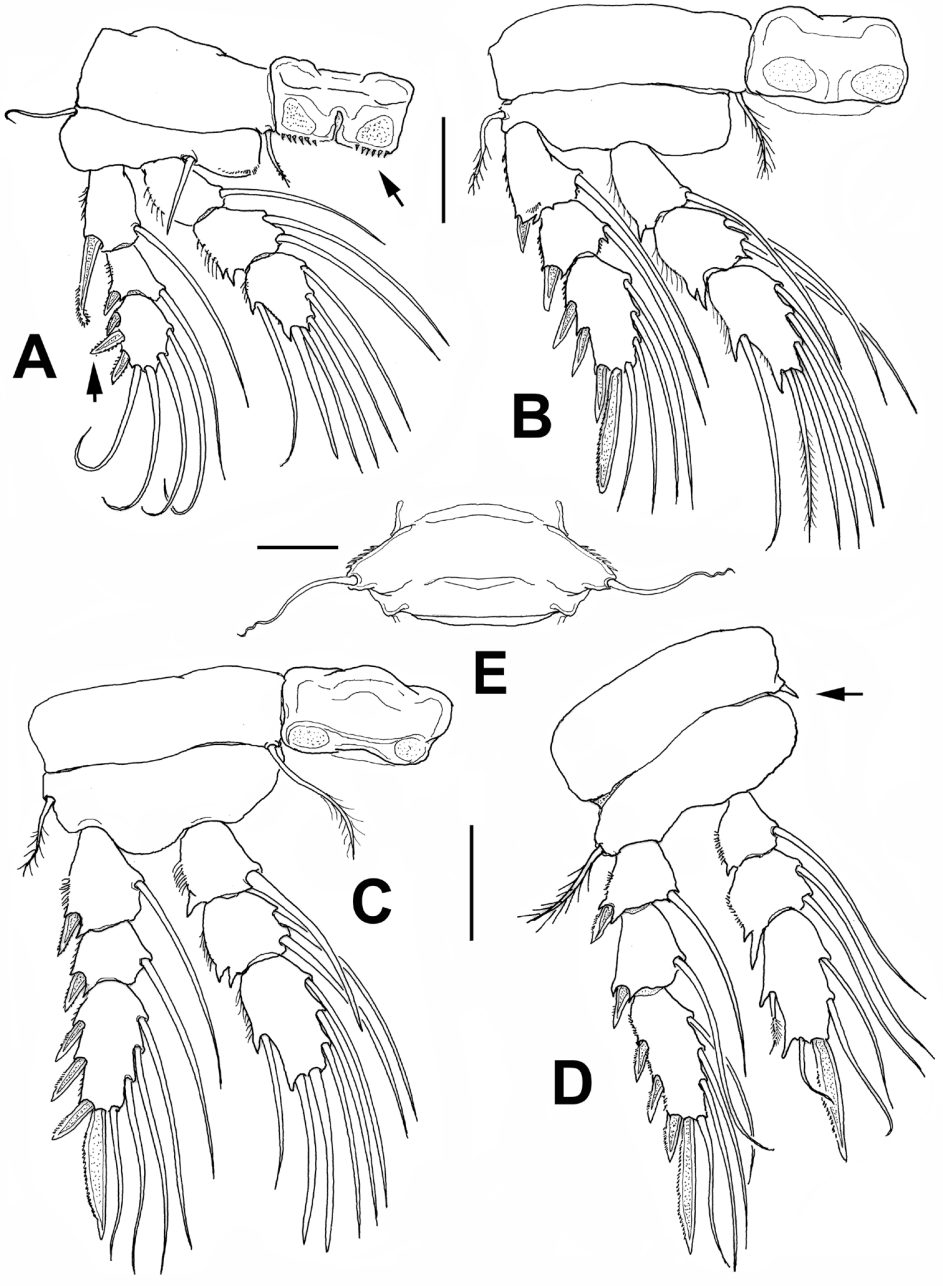
*Monocheres sergioi* sp. n. from Florida. Holotype female. **A** leg 1 **B** leg 2 **C** leg 3 **D** leg 4 **E** fifth pedigerous somite showing reduced fifth leg, ventral view. Scale bars: **A–E** = 50 µm.

*Legs 1– 4* (Fig. 3A–D) biramous, all rami 3-segmented. Coxal sclerites subrectangular, with posterior margins smooth except for leg 1, with row of +10 spinules (arrowed in Fig. 3A). Coxae of legs 1–4 with inner coxal seta; in leg 4 seta reduced, represented by short spiniform element (Fig. 3D). Legs 1–4 with outer basipodal seta; leg 1 bearing short, stout inner basipodal seta. Leg 1 with row of small spinules along inner margin of basis. Outer spine on first exopodal segment of leg 1 strong, with curved tip, reaching insertion of proximalmost spine of third exopodal segment. Medial spine on leg 1 EXP3 being 1.5 times as long as other two spines on same segment (arrowed in Fig. 3A). ENP2 of legs 1–4 with bifurcate projections at outer distal corner; projection longest in leg 4. Leg 4 with reduced outer seta on third endopodal segment.

Spine and setal armature of legs 1–4 as follows:

**Table:** 

Leg	coxa	basis	exopod	endopod
1	0-1	I-1	I-1; I-1;III,1,3	0-1;0-2; 1,2,3
2	0-1	0-1	I-1; I-1; III,I1,3	0-1; 0-2; 1,2,3
3	0-1	0-1	I-1; I-1; III,I1,3	0-1; 0-2; 1,2,3
4	0-I	0-1	I,1; I-1; III,I1,3	0-1; 0-2; 1,1I,2

*Leg 5* (Figs 2A, 3E): strongly reduced, fused to somite; represented by rounded lateral expansion ornamented with row of 6–7 spinules, armed with slender, smooth distal seta, 70 µm long.

**Male.** Unknown.

##### Type locality.

Long Key, Florida Keys, Monroe County, Florida, USA (24.844°N, 80.864°W).

##### Etymology.

The new species is named after Dr. Sergio Salazar Vallejo, senior researcher at El Colegio de la Frontera Sur, for his valuable contributions to the taxonomy and diversity of tropical benthic polychaetes and for finding the copepod specimen herein described.

##### Habitat.

The benthic polychaete containing the copepod, *Hesione picta*, is a widespread species distributed in the western Atlantic Ocean from Florida to Brazil. Locally, it was found in rubble/ sand / seagrass bottom of the type locality. The original host of the copepod remains unknown.

##### Remarks.

The specimen examined was identified as a species of *Monocheres* by its possession of a reduced fifth leg, represented by a papilla-like process arising directly from the somite and armed with a single distal seta. All other characters resemble those known in members of *Asterocheres* (Stock 1966; Kim 2010). The new species can be distinguished from the two other species of the genus, *Monocheres mauritianus* and *Monocheres cagarrensis*, by several differences, as presented in Table 1. Some of the characters used by Johnsson and Bustamante (1997) to compare *Monocheres mauritianus* and *Monocheres cagarrensis* were not included in this analysis because they rely on the accuracy of the observation and even different drawing styles, like the serrate projection of the second exopodal segment of leg 1 or the presence/absence of denticles on the first and second endopodal segments of all swimming legs. Instead, other characters that were deemed stronger were added, like the lack of an inner basipodal spine in *Monocheres cagarrensis* and the presence of spinules along the posterior margin of the coxal sclerite of leg 1. The main apomorphies include the presence of spines along the posterior margin of the first leg coxal sclerite, the shape of the cephalosome, and the reduced, spiniform coxal seta on leg 4. The differences presented in Table 1 serve to clearly distinguish the three species of this genus.

The new species was described based on a single specimen; this is not unusual among the asterocherid copepods; the type species of *Monocheres*, *Monocheres mauritianus*, was also described on a single female specimen collected from the cauliflower coral *Pocillophora damicornis* (L.). This is the third species described in *Monocheres* after its description 50 years ago; there was a 31 year period between the description of the first one, *Monocheres mauritianus*, and the finding of *Monocheres cagarrensis* in Brazil; almost 20 years later a third species was unexpectedly found as described herein.

##### Ecological comments.

Because of the peculiar circumstances by which this specimen was recovered, it is difficult to determine the nature of its association with any of the local benthic invertebrate groups. The associations of asterocherid copepods take place with different invertebrate taxa and the host remains unknown for many species, but asterocherids have not been reported as symbionts of polychaetes (Boxshall and Halsey 2004; Bandera and Huys 2008). These copepods are all ectosymbionts except for *Collocherides astroboae* Stock, 1971, living as an endosymbiont in the stomach of ophiurids. Hence, it is assumed that the hesionid polychaete *Hesione picta*, usually living under rocks, consumed this copepod as a prey or among portions of its food, possibly from sclerobiotic sponges or coral. The copepod remained in the digestive chamber for some time before the fixation of the polychaete and thus, some structures or muscles were expected to be damaged but they were not; the specimen (not an exuvia) was in good condition for taxonomical analysis. It is likely that this individual remained in the caecum for a short time before the polychaete was collected and preserved.

It is interesting to note that *Hesione picta* has been recorded in association with ophiurids living under rocks (De Assis et al. 2012). There are more than 20 known species of asterocherid copepods which are ectosymbiotic in ophiurids, including species of *Asterocheres*, *Collocheres* Canu, 1863, *Collocherides* Stock, 1971, and *Ophiurocheres* (Humes, 1988) (Humes 1998; Doignon et al. 2004), which supports the alternative notion that this copepod was possibly consumed by the polychaete directly from an ophiurid during this hypothetical symbiosis. Hence, the original host of this copepod remains unknown but it is expected that this finding will motivate new observations on these associations involving ophiurids and copepods in the region. A similar situation was reported by Kolbasov et al. (2007); they described a new species of a facetotectan crustacean larva from specimens found together with other food items in the gut of a fish, but in this case the larva is deemed as free-living, with no indication of a symbiotic behaviour.

Other members of the genus *Monocheres* have been known from corals and sponges and only from islands (Stock 1966; Johnsson and Bustamante 1997); this is also the case in the new species, found in the Florida Keys. It is speculated that both isolation and habitat specialization could have a role in the divergence of this genus, with a striking reduction of the fifth leg that strongly diverges from the main asterocherid pattern.

**Table 1.** Comparison of characters of three species of *Monocheres* Based on Johnsson & Bustamante (1997), Stock (1966), and present data.

	*Monocheres mauritianus*	*Monocheres cagarrensis*	*Monocheres sergioi* sp. n.
Pediger 1	posterolateral corners produced, angular	posterolateral corners produced, angular	posterolateral corners not produced, rounded
Pediger 3	posterior margin straight	posterior margin straight	posterior margin weakly curved
Postero-lateral corners of genital double-somite	without processes, rounded	with group of denticles	with acute chitinous projection
Genital openings	with protuberant papilla-like chitinous process and two setae	with cluster of short setules	with low rounded process, two setae and row of setules
Postero-lateral corners of preanal somite	without denticle	with denticle	with denticle
First segment of antennary endopod	shorter than basis	longer than basis	shorter than basis
Exopodal seta of antenna	short	long	long
terminal antennary claw/ first endopodal segment length ratio	1.0	1.5	1.0
Preanal/anal somites length ratio	0.85	1.4	1.0
Posterior margin of anal somite	smooth	smooth	with crenulated hyaline fringe
Inner basipodal spine on leg1	present	absent	present
Posterior margin of leg 1 coxal sclerite	smooth	smooth	with spinules
Coxal seta on leg4	normal	normal	reduced, spiniform
Number of spines on EXP3 of leg 4	3	4	4
Exopodal spines on legs 1-4	shorter than segmental width	longer than segmental width	shorter than segmental width
Exopodal spines on EXP3 of leg 1	distalmost longest	equally long	medial longest
Length of outer lateral seta on ENP3 of leg4	no data available	reaching well beyond distal end of segment	barely reaching distal end of segment

### Key to the species of *Monocheres*

**Table:** 

1	Posterolateral corners of cephalosome with angular corners posteriorly produced, leg 1 without inner basipodal spine, posterolateral corners of genital double-somite with cluster of denticles	***Monocheres cagarrensis* Johnsson & Bustamante, 1997**
–	Posterolateral corners of cephalosome with angular corners rounded or not posteriorly produced, leg 1 with inner basipodal spine, posterolateral corners of genital double-somite smooth or with chitinous projection and setae	**2**
2	Coxal sclerite of leg 1 with smooth posterior margin; coxal seta of normal attributes; distalmost spine on leg 1 third exopodal segment longest	***Monocheres mauritianus* Stock, 1966**
–	Coxal sclerite of leg 1 with spinules along posterior margin; coxal seta reduced, spiniform, medial spine on leg 1 third exopodal segment longest	***Monocheres sergioi* sp. n.**

## Supplementary Material

XML Treatment for Monocheres sergioi

## References

[B1] Bandera ME Huys R (2008) Proposal of a new genus for *Asterocheres mucronipes* Stock, 1960 (Copepoda, Siphonostomatoida, Asterocheridae) an associate of the scleractinian coral *Astroides calycularis* (Pallas, 1766) in the Strait of Gibraltar. Zoological Journal of the Linnean Society 152: 635–653.

[B2] Boxshall GA Halsey SH (2004) An introduction to copepod diversity. The Ray Society, London, 966 pp.

[B3] De Assis JE Bezerra EAS de Brito RJ Gondim AI Christoffersen ML (2012) An association between *Hesione picta* (Polychaeta: Hesionidae) and *Ophionereis reticulata* (Ophiuroidea: Ophionereididae) from the Brazilian Coast. Zoological Studies 51: 762–767.

[B4] Doignon G Deheyn D Fiers F (2004) *Telestacicola xenophiothricis* n. sp. (Copepoda, Poecilostomatoida), a remarkably well adapted commensal of the brittle star *Ophiothrix purpurea* (Echinodermata). Belgian Journal of Zoology 134: 67–73.

[B5] Hartman O (1959) Catalogue of the polychaetous annelids of the world. Allan Hancock Foundation Publications, Occasional Paper 23: 1–628.

[B6] Humes AG (1987) Copepoda (Siphonostomatoida) associated with Ophiuroidea in Jamaica, Puerto Rico, and Barbados. Zoologische Verhandelingen Leiden 323: 365–382.

[B7] Huys R Boxshall GA (1991) Copepod Evolution. The Ray Society, London 159, 468 pp.

[B8] Johnsson R Bustamante AO (1997) *Monocheres cagarrensis* sp. nov. (Copepoda, Siphonostomatoida) from Brazil. Crustaceana 70: 894–900.

[B9] Kim I-H (2010) Siphonostomatoid Copepoda (Crustacea) associated with invertebrates from tropical waters. Korean Journal of Systematic Zoology. Special Issue 8: 1–176.

[B10] Kolbasov GA Grygier MJ Ivanenko VN Vagelli AA (2007) A new species of the y-larva genus *Hansenocaris* Ito, 1985 (Crustacea: Thecostraca: Facetotecta) from Indonesia, with a review of y-cyprids and a key to all their described species. Raffles Bulletin of Zoology 55: 343–353.

[B11] Stock JH (1966) Cyclopoida Siphonostoma from Mauritius (Crustacea, Copepoda). Beaufortia 13(159): 145–194.

[B12] Walter TC Boxshall GA (2016) *Asterocheres* Boeck, 1859. In: Walter TC Boxshall G (Eds) World of Copepods database. Accessed through: World Register of Marine Species at http://www.marinespecies.org/aphia.php?p=taxdetails&id=135554 [on 2016-05-02]

